# The Specific Role of Dermatan Sulfate as an Instructive Glycosaminoglycan in Tissue Development

**DOI:** 10.3390/ijms23137485

**Published:** 2022-07-05

**Authors:** Shuji Mizumoto, Shuhei Yamada

**Affiliations:** Department of Pathobiochemistry, Faculty of Pharmacy, Meijo University, 150 Yagotoyama, Tempaku-ku, Nagoya 468-8503, Japan; shuheiy@meijo-u.ac.jp

**Keywords:** biglycan, carbohydrate sulfotransferase 14, decorin, chondroitin sulfate, dermatan sulfate, dermatan sulfate epimerase, dermatan 4-*O*-sulfotransferase, Ehlers–Danlos syndrome, glycosaminoglycan, proteoglycan

## Abstract

The crucial roles of dermatan sulfate (DS) have been demonstrated in tissue development of the cutis, blood vessels, and bone through construction of the extracellular matrix and cell signaling. Although DS classically exerts physiological functions via interaction with collagens, growth factors, and heparin cofactor-II, new functions have been revealed through analyses of human genetic disorders as well as of knockout mice with loss of DS-synthesizing enzymes. Mutations in human genes encoding the epimerase and sulfotransferase responsible for the biosynthesis of DS chains cause connective tissue disorders including spondylodysplastic type Ehlers–Danlos syndrome, characterized by skin hyperextensibility, joint hypermobility, and tissue fragility. DS-deficient mice show perinatal lethality, skin fragility, vascular abnormalities, thoracic kyphosis, myopathy-related phenotypes, acceleration of nerve regeneration, and impairments in self-renewal and proliferation of neural stem cells. These findings suggest that DS is essential for tissue development in addition to the assembly of collagen fibrils in the skin, and that DS-deficient knockout mice can be utilized as models of human genetic disorders that involve impairment of DS biosynthesis. This review highlights a novel role of DS in tissue development studies from the past decade.

## 1. Introduction

**Figure 1:**
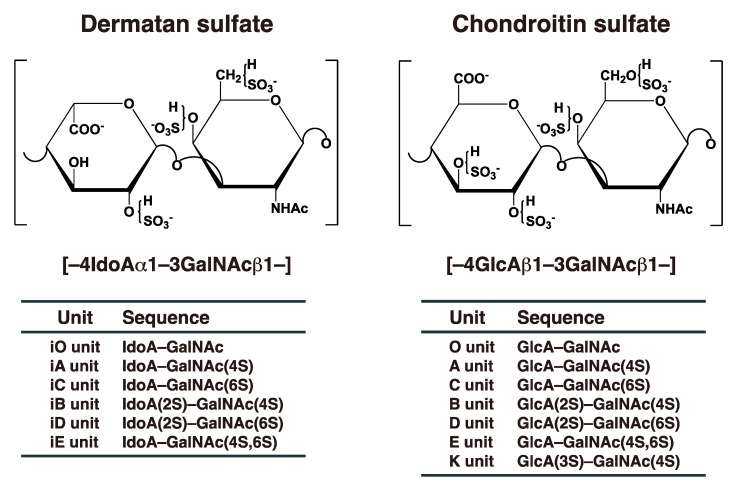
Typical repeating disaccharide units in CS and DS, and their potential sulfation sites. The CS backbone consists of GlcA and GalNAc, whereas DS is a stereoisomer of CS that includes IdoA instead of GlcA. These sugar moieties may be esterified by sulfate at various positions indicated by “SO_3_^–^”. The disaccharide units of CS and DS chains are classified as shown. The abbreviation “i” in DS units stands for IdoA, and 2S, 3S, 4S, and 6S stand for 2-*O*-, 3-*O*-, 4-*O*-, and 6-*O*-sulfate groups, respectively. The representative disaccharide compositions of CS/DS from various tissues and animal species are described in reference [11].

Dermatan sulfate (DS) was initially isolated from skin by Karl Myer in 1941 [1]. Although dermatan sulfate-proteoglycans (DS-PGs) ubiquitously exist in various tissues, they distribute abundantly throughout skin, cartilage, and the aorta [2]. DS-PGs play important roles in anti-coagulation, binding to growth factors, wound healing, in the assembly of extracellular matrices, in tissue morphogenesis, and neuronal homeostasis [3,4,5,6,7,8,9]. DS side chains are linear polysaccharides covalently attached to core proteins that form PGs [2,10]. DS chains consist of alternating disaccharide units comprising *N*-acetyl-D-galactosamine (GalNAc) and L-iduronic acid (IdoA) residues with 50–200 repeats (Figure 1), constructed by specific glycosyltransferases and epimerase [3]. The repeating disaccharide region of DS undergoes maturation by sulfation at the C-4 and C-2 positions on GalNAc and IdoA residues, respectively, thereby exerting a variety of biological functions [3,4,5,6,7,8,9]. It should be noted that chondroitin sulfate (CS) consists of GalNAc and D-glucuronic acid (GlcA) (Figure 1). After biosynthesis of the chondroitin precursor chain, the GlcA residue is epimerized to IdoA by DS-epimerase (DSE). Therefore, the ratio of IdoA to GlcA is distinct, and CS-DS hybrid chains are formed in each organ or developmental stage [3,5,6,8].

Ehlers–Danlos syndrome (EDS) is a heterogenous group of heritable connective tissue disorders characterized by skin hyperextensibility, joint hypermobility, and tissue fragility [12,13]. Various types of EDS are caused by defects in DS in addition to collagens, collagen-modifying enzymes, or Tenascin-X [12,13]. The spondylodysplastic type of EDS that is characterized by kyphoscoliosis, hypermobile joints, generalized osteopenia, short stature, clubfeet, elbow malalignment, muscle hypotonia, wrinkled skin, a characteristic facial appearance, and defective wound healing, is caused by mutations in *B3GALT6* or *B4GALT7* [14,15,16,17,18]. *B3GALT6* and *B4GALT7* encode distinct galactosyltransferase, responsible for glycosaminoglycans including CS, DS, and heparan sulfate (HS) [19,20]. Furthermore, the musculocontractural type of EDS that is characterized by kyphoscoliosis, muscular hypotonia, joint hypermobility, multiple joint contracture, hyperextensible and thin skin, atrophic scars on the skin, characteristic craniofacial features, joint laxities, and recurrent dislocations, is caused by mutations in *DSE* or *CHST14* [21,22,23,24]. *DSE* and *CHST14* encode DSE and dermatan 4-*O*-sulfoteransferase (D4ST), respectively, which function to biosynthesize DS [25,26,27]. Both knockout mice exhibited similar phenotypes such as skin fragility, thoracic kyphosis, and spina bifida, to patients with EDS of the musculocontractural type [28,29,30,31,32]. Therefore, DS is an indispensable macromolecule for the normal development of various tissues. This review focuses on and discusses not only functions of DS, but also DS-defective model animals studied in the past decade.

## 2. Biosynthesis of DS Chains

**Figure 2:**
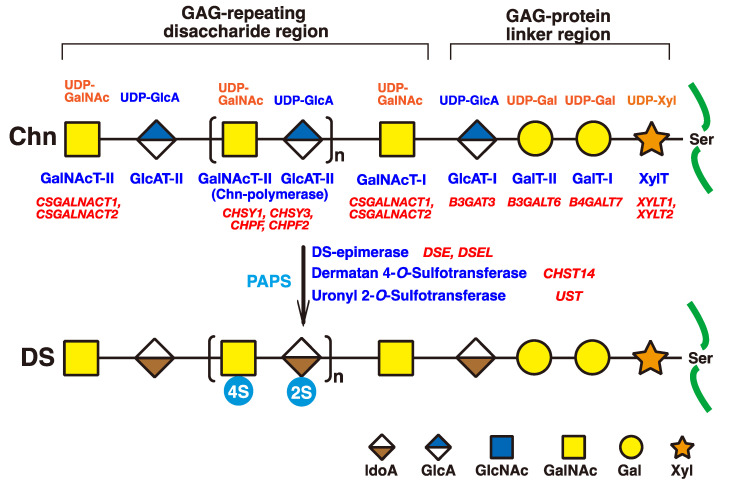
Schematic presentation of biosynthesis of the DS chain. All glycosyltransferases require a corresponding UDP-sugar, such as UDP-Xyl, UDP-Gal, UDP-GlcA, and UDP-GalNAc, as a donor substrate. After specific core proteins have been translated, the GAG-protein linker region, GlcA-Gal-Gal-Xyl-, is constructed by XylT, GalT-I, GalT-II, and GlcAT-I. The fifth sugar moiety, GalNAc, is then transferred to the GlcA residue in the linker region by GalNAcT-I, thereby resulting in the formation of the repeating disaccharide region, [-GlcA-GalNAc-]_n_, which is the unsulfate backbone of CS, by Chn-polymerase that is formed by a hetero complex of any CHSY1, CHSY3, CHPF, or CHPF2. Then, DS-epimerase converts GlcA into IdoA by epimerizing the C-5 carboxy group in the chondroitin precursor, which results in the formation of the repeating disaccharide region of dermatan, [-IdoA-GalNAc-]_n_. After formation of the dermatan backbone, each sugar residue is modified by sulfation and catalyzed by sulfotransferases, as indicated in the figure. D4ST or UST transfers a sulfate group from PAPS to the C-4 position of the GalNAc or to the C-2 position of the IdoA residues in the dermatan chain, respectively. It should be noted that the 4-*O*-sulfation but not the 2-*O*-sulfation is predominant. Each enzyme and its coding gene are described under the respective sugar symbols. The abbreviations 2S and 4S stand for 2-O- and 4-*O*-sulfates, respectively.

The initiation of GAGs is evoked by the transfer of D-xylose (Xyl) from uridine diphosphate (UDP)-Xyl to specific serine residues in the core proteins of PGs by xylosyltransferase (XYLT) in the endoplasmic reticulum and cis-Golgi compartments (Figure 2) [33,34]. Subsequently, two galactoses (Gals) and a GlcA residue are transferred from UDP-Gal and UDP-GlcA by galactosyltransferases-I (GalT-I) [19], GalT-II [20], and glucuronyltransferase-I (GlcAT-I) [35], respectively, which results in the formation of the common glycosaminoglycan–protein linker region tetrasaccharide, GlcA-Gal-Gal-Xyl-*O*- (Figure 2) [36,37].

The chondroitin precursor chain, [-4GlcAβ1-3GalNAcβ1-]_n_, which is the unsulfate backbone of CS, is built up to the non-reducing terminal GlcA residue of the linker region tetrasaccharide by chondroitin synthase family members, using UDP-GlcA and UDP-GalNAc as the donor substrate (Figure 2) [38,39,40,41,42,43]. The GlcA residues in [-4GlcAβ1-3GalNAcβ1-]_n_ are converted into IdoA by epimerizing the carboxy group of GlcA by DSE during and/or after the formation of a chondroitin backbone [25,44], resulting in formation of the repeating disaccharide region of dermatan, [-4IdoAα1-3GalNAcβ1-]_n_. D4ST1 and UST transfer the sulfate group from the sulfate donor 3′-phosphoadenosine 5′-phosphosulfate (PAPS) to the C-4 position of GalNAc and to the C-2 position of IdoA residues in dermatan, respectively [26,27,45].

## 3. Classical and Additional Functions of DS

### 3.1. Classical Functions of DS

Although classical functions of DS have been described in the literature [4,10,46], they are briefly introduced in this section. The DS side chain of decorin binds to collagen to assemble the extracellular matrix [47,48] (Table 1). A focused ion beam scanning electron microscope revealed that the DS side chain of decorin forms a ring-mesh like structure, with each ring surrounding a collagen fibril [49,50].

Heparin cofactor II, like antithrombin III, inhibits proteolytic enzymes involved in blood coagulation via interaction with DS as well as heparin [51] (Table 1). The DS-derived hexasaccharide, [-IdoA(2-*O*-sulfate)-GalNAc(4-*O*-sulfate)-]_3_, from porcine skin has been identified as the smallest fragment of DS binding to heparin cofactor II with high affinity [52].

DS regulates specific cell signaling through interactions with effector molecules such as fibroblast growth factors (FGFs) and hepatocyte growth factor (HGF) [53,54,55] (Table 1). The minimum sizes for cell proliferation through FGF2 as well as FGF7 are octa- and decasaccharides, respectively [56]. Furthermore, the disulfated disaccharide unit, [-IdoA(2-*O*-sulfate)-GalNAc(4-*O*-sulfate)-], from Ascidian, *S. plicata*, shows greater activity than the monosulfated disaccharide unit, [-IdoA-GalNAc(4-*O*-sulfate)-] from porcine intestinal mucosa [56].

Highly sulfated DS containing characteristic disaccharide units such as [-IdoA(2-*O*-sulfate)-GalNAc(4-*O*-sulfate)-], [-IdoA(2-*O*-sulfate)-GalNAc(6-*O*-sulfate)-], or [-IdoA-GalNAc(4-*O*-, 6-*O*-disulfates)-] derived from Ascidian (*S. plicata* and *A. nigra*), embryonic sea urchin, notochord of hagfish, or shark skin, exerted neurite outgrowth-promoting activity of hippocampal neurons in vitro [57,58,59] (Table 1). The activity might be mediated by neurotrophic factors including pleiotrophin, brain-derived neurotrophic factor (BDNF), glial cell line-derived neurotrophic factor (GDNF), HGF, and/or FGFs [59,60,61].

DS and/or DS-PGs are up-regulated in tumor cells as well as in the stroma [62,63,64,65], which is consistent with up-regulations of glycosyltransferases, epimerases, and sulfotransferases responsible for the biosynthesis of DS [65]. Furthermore, IdoA-deficient human esophagus squamous cell carcinoma by shRNA showed decreased migration and invasion capabilities in vitro, which was associated with reduced cellular interaction with HGF, inhibition of pERK-1/2 signaling, and deregulated actin cytoskeleton dynamics and focal adhesion formation [65]. These findings suggest that DS and/or DS-PGs may contribute to proliferation, invasion, and metastasis via binding with effector proteins. However, what remains unclear is the ratio of CS/DS, the content of IdoA, chain length, sulfation pattern, binding molecules, and cell signaling. Further studies are required in order to clarify the molecular mechanisms involving DS-PGs through the use of model animals as well as clinical specimens.

**Table 1:** Biological activities of a variety of DS variants.

DS Origin	Molecular Weight	IdoA Content	Binding Protein(s)	Biological Activities	Reference
Porcine skin	11–25 kDa	~75%	Heparin cofactor II, FGF2, FGF7, collagen	Anti-coagulation, cell growth, assembly of extracellular matrix	[2,47,51,66]
Ascidian (*A. nigra*)	––	~100%	Heparin cofactor II	Anti-coagulation, neurite outgrowth-promoting activity	[57,67]
Ascidian (*S. plicata*)	––	~70%	Heparin cofactor II	Anti-coagulation, neurite outgrowth-promoting activity	[57,68]
Embryonic sea urcin	––	~100%	––	Neurite outgrowth-promoting activity	[57,69]
Hagfish notochord	18 kDa	60~75%	FGF2, FGF10, FGF16, FGF18, Midkine, Pleiotrophin, Heparin-binding EGF-like growth factor (HB-EGF), Vascular endothelial growth factor (VEGF), BDNF, GDNF	Neurite outgrowth-promoting activity	[58]
Shark skin	70 kDa	42%	FGF2, FGF10, FGF16, FGF18, Midkine, Pleiotrophin, HB-EGF, VEGF, BDNF, GDNF, heparin cofactor II	Neurite outgrowth-promoting activity, anti-coagulation	[59,61,70]

––, not reported.

### 3.2. Recent Additional Functions of DS

Recently, it was shown that molecules longer than tetrasaccharides derived from DS enhance the activation of anaplastic lymphoma kinase, a receptor tyrosine kinase, by clustering of anaplastic lymphoma kinase [62].

Furthermore, it was demonstrated that the expressions of *Dsel* and *D4st1* increased during formation of the embryonic body from mouse embryonic stem cells, and that an addition of DS to the culture medium promoted neuronal differentiation by activation of extracellular signal-regulated kinase 1/2, and also accelerated neurite outgrowth in mouse embryonic stem cells [71]. On the other hand, knockdown or overexpression of D4ST1 in mouse embryonic stem cells led to the promotion or suppression of endodermal differentiation, respectively [72]. These opposite effects of the addition of DS as well as knockdown or overexpression of D4ST1 on differentiation of mouse embryonic stem cells remain unclear; further study is required to explain these phenomena. In addition, DS promoted neuronal differentiation and neuronal migration, but not neurite outgrowth in human neuronal stem cells [71]. These findings indicate that DS may modulate neuronal differentiation in both mouse and human stem cells.

Although knockdown of C4ST1 by antisense morpholino oligonucreotide accelerated regeneration of axons after spinal cord injury in zebrafish, knockdown of D4ST1 did not [73], indicating that 4-*O*-sulfation of CS, but not DS, inhibit axonal regrowth after spinal cord injury.

## 4. Knockout and Mutant Mice of Biosynthetic Enzymes of DS

### 4.1. Dse

In 2009, Maccarana and coworkers generated *Dse* knockout mice. Their studies showed that the knockout mice of *Dse* were smaller, with a 20–30% reduced body weight at birth compared with that of wild-type mice [28] (Table 2). All *Dse^–/–^* pups had kinked tails until 4 weeks of age, and *Dse^–/–^* mice showed reduced fertility. Histological analysis of the skin from *Dse^–/–^* mice exhibited sparser loose connective tissue in the hypodermal layer compared with that of wild-type mice. However, the distributions and amounts of core proteins, decorin and biglycan, that are DS-PGs, did not change in the skin of *Dse^–/–^* mice. The mean diameters of collagen fibrils from skin were 60 and 85 nm in wild-type and *Dse^–/–^* mice, respectively. Accordingly, the stress at failure was reduced in the skin of *Dse^–/–^* mice. In decorin-deficient mice, which were generated by Iozzo’s group, the collagen fibrils showed irregular outlines with larger diameters than wild-type skin [74]. Thus, DS as well as DS-PGs play a role in skin development. On the other hand, the histological phenotypes of adrenal glands, brains, intestines, kidneys, and lungs in *Dse^–/–^* mice were not detected. Further analyses are required to expand the findings on DS functions.

Epimerase activity of the skin, spleen, lung, and kidney in *Dse^–/–^* mice was markedly lower compared with that of wild-types, whereas its activity in the brain was comparable between *Dse^+/+^* and *Dse^–/–^* mice [28]; this was consistent with their mRNA levels [75,76]. The remaining activity can most likely be attributed to DSE2 encoded by *Dsel*. Furthermore, IdoA-containing structures in the whole body as well as in the skin of 10-day-old pups were markedly reduced in the *Dse^–/–^* mice compared with that of wild-type mice. These findings indicate that DSE and the DS side chains of PGs contribute to the DS biosynthesis and formation of collagen bundles, respectively, in the skin.

**Table 2:** DS-deficient mice and human disorders.

Coding Genes	Phenotypes of Knockout or Mutant Mice	Human Genetic Disorders (MIM Numbers)	Ref. for Knockout Mice	Ref. for Human Disorders
*Dse*	Thicker collagen fibrils in the dermis and hypodermis, smaller body weight, kinked tail, defects in fetal abdominal wall, exencephaly, and spina bifida.	Ehlers–Danlos syndrome musculocontractural type 2 (615539, 605942)	[28,77]	[21,81,82,83,84]
*Dsel*	Normal extracellular matrix features in the brain.	Bipolar disorder; depressive disorder; diaphragmatic hernia; microphthalmia (611125)	[77,78]	[75,85,86]
*Chst14*	Increased skin fragility, disorganized collagen fibers, thoracic kyphosis, reduced fertility, kinked tail, myopathy-related phenotypes such as variation in fiber size and spread of the muscle interstitium, smaller body mass, alterations in the vascular structure of the placenta, an abnormal structure of the basement membrane of capillaries in the placental villus, better recovery after femoral nerve injury.	Ehlers–Danlos syndrome musculocontractural type 1; Ehlers–Danlos syndrome, type VIB; adducted thumb-clubfoot syndrome (601776, 608429)	[29,30,31,32,79,80]	[22,23,24,81,87,88,89,90,91,92,93,94,95,96]

### 4.2. Dsel

In 2015, Maccarana and coworkers generated *Dsel* knockout mice [77,78]. Their studies showed that the *Dsel^–/–^* mice were fertile, and that there were no significant differences in the weight or length at weaning and at 9 weeks of age between wild-type and *Dsel^–/–^* mice [78] (Table 2). Furthermore, *Dsel^–/–^* mice had no anatomical or histological defects in the brain, heart, kidney, liver, lungs, lymph nodes, muscles, pancreas, skin, small intestines, spleen, stomach, or thymus compared with wild-type mice; however, total DS-epimerase activity in the brain, kidney, spleen, liver, lung, and skin was reduced to 11, 45, 56, 62, 66, and 76%, respectively, compared with wild-type mice. Consistently, IdoA contents in CS/DS of brain and kidney from 3-day-old *Dsel^–/–^* were reduced to 62 and 87%, respectively, compared with wild-type mice. Normal extracellular matrix features were detected in the brain section from *Dsel^–/–^* mice compared with wild type. Thus, further behavioral analyses are needed, such as an open-field test, a separation-reunion test, resident-intruder test, and social dominant tube test, in order to explore the functions of DS in the brain, since it has been reported that various mutations in *DSEL* cause bipolar disorder [75].

### 4.3. Dse/Dsel

Double knockout mice of *Dse* and *Dsel*, which were generated by Maccarana’s group in 2015, showed complete loss of the IdoA residue of CS/DS in 2-day-old pups [77]. Total levels of CS/DS disaccharides from various organs on embryonic (E) day 19.5 in *Dse^–/–^/Dsel^–/–^* were reduced to 30% compared with *Dse^+/+^/Dsel^+/+^*. *Dse^–/–^/Dsel^–/–^* embryos showed Mendelian ratios on E13.5–E19.5, and exhibited the defects of abdominal wall closure, a kinked tail, and exencephaly in varying ratios with each phenotype, ranging macroscopically from normal to severely affected. The major organs including the brain, spinal cord, liver, and lung from *Dse^–/–^/Dsel^–/–^* embryos appeared normal when compared with control embryos, *Dse^+/+^/Dsel^–/–^*. Nonetheless, all newborns died in the 48 h after birth, even in the absence of a phenotype, which may be due to multiple fragility and multiple organ failure. Further investigation using this double knockout mouse model is needed to reveal the mechanism of neonatal lethality.

### 4.4. Chst14

In 2011, Schachner and coworkers generated *Chst14* knockout mice [29,79]. Their studies showed that the *Chst14^–/–^* mice were fertile, but that one-third of the knockout mice died between E16.5–E18.5 and/or within a few days after birth [29] (Table 2). The body weight as well as weight of the tibia, heart, liver, and kidney, but not brain, were decreased in the *Chst14^–/–^* mice compared with their wild-type littermates. These observations are partially similar to those of humans with mutations in *CHST14* [22], and indicate that Chst14 and/or DS may be indispensable for early tissue development. DS binds to FGFs as well as HGF [55,56]. Thus, defects in forms of signaling may lead to tissue development in *Chst14^–/–^* mice.

In 2018, Yoshizawa et al. demonstrated that the placenta derived from *Chst14^–/–^* mice exhibited an alteration in the vascular structure with ischemic and/or necrotic-like change, an abnormal structure of the basement membrane of capillaries in the placental villus, a reduced weight of the placenta, and significantly decreased DS [80]. These findings offer further evidence to support the perinatal lethality caused by a defect in *CHST14* [22], and suggest why DS may be essential for placental vascular development and/or assembly of collagen fibrils by DS.

*Chst14^–/–^* mice exhibited elephant teeth, a kinked tail, increased skin fragility, and thoracic kyphosis, compared with *Chst14^+/+^* mice [29,31]. Moreover, Nitahara-Kasahara et al. demonstrated that decreases in DS in muscle in addition to myopathy-related phenotypes such as variation in fiber size and spread of the muscle interstitium which caused lower grip strength and decreased exercise capacity, were observed in *Chst14^–/–^* mice compared with *Chst14^+/+^* as well as *Chst14^+/–^* mice [31,32].

In 2021, Hirose et al. demonstrated that the skin tensile strength of *Chst14^–/–^* mice was lower than that of *Chst14^+/+^* mice, which may be most likely caused by abnormal collagen fibrils in the reticular layer [30]. A DS-PG, decorin-PG, regulates collagen fiber formation [74]. Although DS with a round conformation wrapped the collagen fibrils in the wild-type mice, rod-shaped linear DS was detected at one end of collagen fibrils and protruded outside the fibrils in *Chst14^–/–^* mice [30]. These observations suggest that the DS side chain of decorin is essential for the assembly of collagen and for supporting skin strength, indicating that *Chst14^–/–^* mice is a good model for musculocontractural Ehlers–Danlos syndrome caused by mutations in *CHST14* [22,23,24,87,88].

Bian et al. demonstrated that neurospheres from *Chst14^–/–^* mice showed larger diameters and fewer total numbers than those from *Chst14^+/+^* mice [79]. This was caused by dysfunctions in proliferation and self-renewal of neural stem cells in vivo [79], indicating that DS and/or DS-PGs play important roles in the proliferation and differentiation of neural stem cells. *Chst14^–/–^* mice exhibited longer cell processes as well as a higher proliferation rate of cultured Schwann cells from dorsal roots and nerves, in addition to longer neurites of cultured neurons from cerebella compared with those of *Chst14^+/+^* mice [29]. After femoral nerve injury, *Chst14*-deficiency accelerates the recovery of motor functions, whereas there were no significant differences in the motor reinnervation pattern, degree of myelination, or Schwann cell proliferation between *Chst14^+/+^* and *Chst14^–/–^* mice. Therefore, further analysis may be needed to understand the molecular mechanism underlying motor recovery by *Chst14*-ablation.

## 5. Human Disorders Affecting the Skeleton and Skin Caused by Disturbances in DS Biosynthetic Enzymes

### 5.1. DSE

DSE encoded by *DSE* converts GlcA into IdoA in the chondroitin precursor chain, which results in the formation of dermatan polysaccharide (Figure 2). [25]. EDS musculocontractural type 2 is caused by pathogenic variants in *DSE* including p.Ser268Leu, p.Arg267Gly, p.Tyr320*, p.Val333Cysfs*4, p.Pro384Trpfs*9, p.His588Arg, and p.Val938Asp [21,81,82,83,84] (Table 2). The characteristic manifestations of the EDS musculocontractural type 2 were hypermobility of the finger, elbow, and knee joints, contracture of the thumbs and feet, atrophic scars on the skin, and characteristic facial features such as midfacial hypoplasia, blue sclera, and hypertelorism [21,81,83]. Recombinant DSE-p.Ser268Leu exhibited a loss of epimerase activity [21]. Furthermore, skin fibroblasts from a patient with a mutation of p.Ser268Leu in DSE showed markedly decreased epimerase activity, resulting in a lower level of DS compared with a healthy subject (Table 3) [21]. These findings suggest that these pathogenic variants in *DSE* may cause a defect in DS, which leads to EDS musculocontractural type 2.

### 5.2. DSEL

DSE2/DSEL encoded by dermatan sulfate epimerase-like (*DSEL*) converts GlcA into IdoA in chondroitin, resulting in the formation of dermatan polysaccharide (Figure 2). [44]. *DSEL* was predominantly expressed in the brain [75,78,97]. A bipolar disorder was caused by a variety of homozygous and heterozygous variants in *DSEL* including the substitution of a nucleotide in the 5′-non-coding region, p.Val287Ile, p.Pro673Ser, p.Tyr730Cys, p.Pro942Ser, and p.Ile1113Met [75] (Table 2). Furthermore, single nucleotide polymorphism (SNP), rs17077540, detected at 75 kbp upstream of *DSEL*, causes a recurrent early-onset major depressive disorder [85]. These findings indicate that brain DS and/or DS-PGs produced by DSEL may play a role in the development, homeostasis, and/or function of the central nervous system. A model organism(s) with these variants in *DSEL* might help explore the pathogenic mechanisms of this disorder.

The heterozygous variant, p.Met14Ile, in DSEL causes a late-presenting, anterior diaphragmatic hernia [86] (Table 2). However, it remains unclear how DSEL, DS, or DS-PGs contribute to the development and/or regulation of the diaphragm.

### 5.3. CHST14

D4ST encoded by carbohydrate sulfotransferase 14 (*CHST14*) catalyzes the transfer of a sulfate group from 3′-phosphoadenosine 5′-phosphosulfate to the C-4 hydroxy group of GalNAc residues in DS chains (Figure 2) [26,27]. EDS musculocontractural type 1 is caused by a variety of pathogenic variants in *CHST14* [22,23,24,81,87,88,89,90,91,92,93,94,95,96] (Table 2). The characteristic manifestations of the EDS musculocontractural type 1 were craniofacial features including large fontanelle with delayed closure, downslanting palpebral fissures, and hypertelorism; skeletal features including characteristic finger morphologies, joint hypermobility, multiple congenital contractures, progressive talipes deformities, and recurrent joint dislocation; cutaneous features including hyperextensibility, fine/acrogeria-like/wrinkling palmar creases, and bruisability; refractive errors of vision, large subcutaneous hematomas, constipation, cryptorchidism, hypotonia, and motor developmental delay [96]. Skin fibroblasts from two patients with mutations of p.Pro218Leu/Tyr293Cys and p.Pro218Leu/Pro218Leu in *CHST14* showed markedly decreased sulfotransferase activity, resulting in the loss of DS accompanied with replacement by CS [23] (Table 3). Furthermore, the dispersed collagen fibrils were detected in the papillary to reticular dermis from patients with EDS musculocontractural type 1, compared with the tightly assembled fibrils in healthy controls [50]. This is caused by an affected GAG chain, CS, on decorin with a structurally linear polysaccharide, in contrast to normal DS with a structurally curved polysaccharide. These findings indicate that DS, but not CS, is essential for assembly of the collagen fibril network in connective tissues.

## 6. Conclusions and Perspectives

Mice deficient in DS demonstrated replacement with CS and anomalies of the skin, skeleton, nervous system, and early development, which might be mainly caused by disturbances in collagen bundle formation and in the signaling pathway(s). These findings indicate that interaction of DS with collagens as well as growth factors is dependent on the conformation of the IdoA-containing structure in DS, in a manner similar to heparin. Furthermore, the human genetic disorder Ehlers–Danlos syndrome, caused by a defect in DS, has also been revealed. DS-deficient model mice may become powerful tools in the development of new therapeutics such as applications of mimetics of DS-oligosaccharide, enzyme-replacement therapy, or adeno-associated virus for Ehlers–Danlos syndrome caused by a defect in DS.

## Figures and Tables

**Table 3:** Alteration of CS/DS disaccharides in the skin fibroblasts from patients with mutations in *DSE* and *CHST14*.

Affected Genes	DS	CS	Reference
*DSE*	9% *	70%	[21]
*CHST14*	Not detected	189%	[23]

* Compared with the healthy subjects.

## Data Availability

Not applicable.
